# Experiences from treatment-predictive *KRAS *testing; high mutation frequency in rectal cancers from females and concurrent mutations in the same tumor

**DOI:** 10.1186/1472-6890-9-8

**Published:** 2009-10-15

**Authors:** Mats Jönsson, Anna Ekstrand, Thomas Edekling, Jakob Eberhard, Dorthe Grabau, David Borg, Mef Nilbert

**Affiliations:** 1 Department of Oncology, Institute of Clinical Sciences, Lund university, Lund, Sweden; 2 Department of Oncology, Växjö Hospital, Växjö, Sweden; 3 Department of Pathology, Institute of Clinical Sciences, Lund university, Lund, Sweden; 4 Clinical Research Centre, Hvidovre Hospital, Copenhagen University, Hvidovre, Denmark

## Abstract

**Background:**

*KRAS *mutations represent key alterations in colorectal cancer development and lead to constitutive EGFR signaling. Since EGFR inhibition represents a therapeutic strategy in advanced colorectal cancer, *KRAS *mutation analysis has quickly been introduced as a treatment-predictive test.

**Methods:**

We used a real-time PCR based method to determine *KRAS *mutations in 136 colorectal cancers with mutations identified in 53 (39%) tumors.

**Results:**

*KRAS *mutations were significantly more often found in rectal cancer (21/38, 55%) than in colon cancer (32/98, 33%) (P = 0.02). This finding was explained by marked differences mutation rates in female patients who showed mutations in 33% of the colon cancers and in 67% of the rectal cancers (P = 0.01). Concurrent *KRAS *mutations were identified in three tumors; two colorectal cancers harbored Gly12Asp/Gly13Asp and Gly12Cys/Gly13Asp and a third tumor carried Gly12Cys/Gly12Asp in an adenomatous component and additionally acquired Gly12Val in the invasive component.

**Conclusion:**

The demonstration of a particularly high *KRAS *mutation frequency among female rectal cancer patients suggests that this subset is the least likely to respond to anti-EGFR therapies, whereas the observation of concurrent *KRAS *mutations imply that repeated *KRAS *targeting may occur during tumor progression in a subset of colorectal cancers.

## Background

Inhibition of the epidermal growth factor receptor (EGFR) signaling pathway represents a therapeutic option in advanced colorectal cancer. Improved response rates and prolonged time to metastasis/survival has been demonstrated with the currently registered EGFR blocking antibodies cetuximab and panitumumab, and additional EGFR inhibitors are in various stages of clinical trials. Mutations in the *KRAS *oncogene typically occur already in the late adenoma stage and have since long been recognized as a key event in colorectal cancer development [1]. Overall, *KRAS *mutations are found in about 40% of the tumors and are predominantly located in codons 12 (82% of the mutations reported) and 13 (17%) . Activating mutations lead to permanently GTP bound KRAS and constitutive downstream pathway signaling, also in the absence of upstream EGFR stimulation. Presence of *KRAS *mutations therefore represents a negative predictor of response to EGFR therapy and *KRAS *mutation testing has rapidly moved into the molecular diagnostic work-up of colorectal cancers considered for EGFR treatment [2,3]. Quality assurance programs for *KRAS *mutation testing and practice guidelines related to e.g. optimal testing material, methodological considerations and recommendations for the reporting of the results are currently being developed [4]. PCR-based assays constitute the cornerstone for clinical *KRAS *testing since these analyses allow high-throughput testing and have a favorable sensitivity, also in samples with low tumor cell content. We report the experiences from our first 136 treatment-predictive *KRAS *tests and herein report significant differences in mutation frequencies in colon cancer and rectal cancer and coexisting *KRAS *mutations in a subset of the tumors.

## Methods

*KRAS *mutation testing was performed in 136 adenocarcinomas of the colon (n = 98) and the rectum (n = 38). The mean age was 56 (21-81) years and the series included 64 (47%) females. Representative tumor blocks were selected and were in the majority of the cases derived from the primary tumor, in 13 cases from metastatic tissue and in four cases from a local recurrence. Presence of at least 20% tumor cells in the tissue was verified by a pathologist. DNA was extracted from serial sections of formalin-fixed, paraffin-embedded tumor tissue using the Qiamp DNA FFPE tissue Kit (Qiagen, Hilsen, Germany) according to the manufacturer's recommendations. Standard clinical analysis applied the DxS real-time PCR based kit (Roche Diagnostics, Basel, Switzerland), which identifies 7 different *KRAS *mutations in exons 12 and 13 with high sensitivity. In order to confirm presence of double or triple coexisting mutations, samples with such unusual patterns were also subjected to pyrosequencing (PyroMark™ Q96 KRAS v2.0, Qiagen, Hilsen, Germany) according to the manufacturer's recommendations on PSQ™HS96A [5]. Mutations were quantified using the machine's software. Statistical analysis used a Chi2 test and the level of significance was set at 5%. *KRAS *mutation testing was carried out as part of standard care, all patients provided informed consent for testing, and the study was conducted according to the Helsinki declaration.

## Results

*KRAS *mutations were identified in 53/136 (39%) colorectal cancers. Overall, mutation status did not correlate with sex when analyzed in the whole cohort with mutations in 28/64 (44%) women and in 5/72 (35%) men (P = 0.28) or age (mean age 55 years in the mutant group and 57 years in the wild-type group). KRAS mutations, however, significantly correlated with tumor location with mutations found in 32/98 (33%) colon cancers and 21/38 (55%) rectal cancers (P = 0.02; Chi2-test). This difference was related to a high mutation rate in female rectal cancer patients (14/21, 67%) compared to females with colon cancer (14/43, 33%) (p = 0.01), whereas no significant difference related to tumor location was identified in men (41% in rectal cancer and 33% in colon cancer, P = 0.52). Though the materials in subsets are small, women with KRAS mutant rectal cancers were diagnosed mean 10 years earlier (mean age 48, range 25-71, years) than those with *KRAS *wild-type rectal cancers (mean age 58, range 52-70, years).

**Figure 1:**
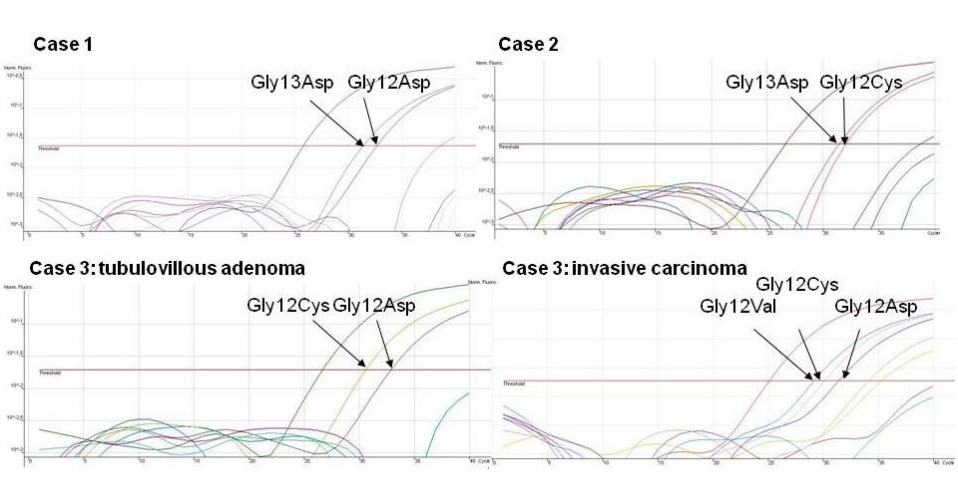
**Real-time PCR analysis identified coexisting KRAS mutations in three cases**. Case 1) colon cancer with the Gly12Asp and Gly13Asp mutations, Case 2) colon cancer with coexisting Gly12Cys and Gly13Asp mutations, and Case 3) a rectal tumor that revealed Gly12Asp and Gly12Cys in adenomatous components and an additional Gly12Val mutation in the invasive tumor component. All mutations were verified using pyrosequencing. Case 1 contained 12% Gly12Asp and 24% Gly13Asp, and case 2 4% Gly12Cys and 34% Gly13Asp. Case 3 harboured 17% Gly12Asp and 22% Gly12Cys in the adenomatous component, and 10% Gly12Asp, 7% Gly12Cys, and 12% Gly12Val in the invasive component (data not shown).

The mutation spectrum revealed the expected codon 12 and 13 mutations with the p.Gly12Asp, p.Gly13Asp, and p.Gly12Val being the most commonly found (table 1) [6]. Three tumors showed two-three different *KRAS *mutations (figure 1), which were identified using real-time PCR and verified in a separate tumor block and using pyrosequencing as a complementary method. The first case was a colon cancer from a 65-year old man with hereditary nonpolyposis colorectal cancer and a germline mutation in the *MLH1 *gene. The tumor was found to harbor two *KRAS *mutations, Gly12Asp (ΔCT value 6.9, cutoff 8) and Gly13Asp (ΔCT value 5.6, cutoff 9). The patient had 10 years earlier developed two synchronous colon cancers, both of which were retrospectively analyzed, but neither of these contained any *KRAS *mutation (data not shown). The second case was a 68-year old man with a rectal cancer metastatic to the liver. The primary tumor revealed coexisting coexistence of the *KRAS *mutations Gly12Cys (ΔCT value 5.4, cutoff 7.0) and Gly13Asp (ΔCT 4.8, cutoff 9.0). The third case was a 47-year old woman who presented with a rectal cancer and concurrent liver metastases. The initial biopsy from the rectal tumor revealed only adenomatous components, whereas a second biopsy contained infiltrative adenocarcinoma. Unfortunately, no histologic sample from the liver lesions was available. The tubulovillous adenoma harbored the mutations Gly12Asp (ΔCT value 6.2, cutoff 8.0) and Gly12Cys (ΔCT value 3.9, cutoff 7.0), whereas the sample with also an invasive component contained both these mutations and in addition (contained) a Gly12Val mutation (ΔCT value 4.1, cutoff 6.5). All mutations were identified by real-time PCR and verified using pyrosequencing.

**Table 1:** Summary of *KRAS *mutations identified.

***KRAS *mutation**	**Amino acid change**	*Number (%) mutations	**Distribution colon/rectum**
c.35G>A	p.Gly12 Asp	19 (33)	13/6
c.38G>A	p.Gly13Asp	12 (21)	8/5
c.35G>T	p.Gly12Val	10 (18)	4/6
c.34G>T	p.Gly12Cys	7 (12)	6/1
c.34G>A	p.Gly12Ser	5 (9)	1/4
c.35G>C	p.Gly12Ala	4 (7)	3/1
c.34G>C	p.Gly12Arg	-	-

*Represents 53 tumors, including 3 with coexisting mutations

## Discussion

The overall *KRAS *mutation frequency of 39% is in line with the results from previous studies , though the different mutation rates - 33% in colon cancer and 55% in rectal cancer (p = 0.02) - has not been reported in conjunction with treatment-predictive testing. Distinct molecular pathways are preferentially involved in the adenoma-carcinoma sequence in the proximal and the distal colon with *TP53 *and *KRAS *mutations being favored in rectal cancer, whereas mismatch repair defects are more common in proximal colon tumors [7]. A higher mutation frequency in rectal cancers compared to colon cancer has previously been described, although the magnitude has been lower than identified herein [8]. The effect seems to be limited to females with rectal cancer, 67% of whom harbored *KRAS *mutations. This observation is in line with the findings in a large population-based series from the Netherlands in which Brink et al. reported a higher *KRAS *mutation rate in female rectal cancer patients [8]. Furthermore, in the subset of females with rectal cancer the KRAS mutant tumors were diagnosed mean 10 years earlier (mean age 48 years) behaviour than the wild-type tumors (mean age 58). Though this observation is new and needs confirmation, a recent study has demonstrated that early-onset colorectal cancers frequently show pathologic features associated with aggressive clinical features [9]. The tumors here investigated were selected based on potential EGFR treatment, which may bias the results toward tumors prone to metastatic development, though the overall *KRAS *mutation frequency of 39% and the relation between colon cancer and rectal cancer are as expected. The differences in *KRAS *mutation rates identified suggest that sex and tumor location influence the chance of clinical usefulness of EGFR inhibitors with a particularly low likelihood of benefit among female rectal cancer patients.

A stepwise accumulation of mutations in different pathways constitutes a hallmark of colorectal cancer development. The observation of coexisting *KRAS *mutations in a small number of colorectal cancers, however, suggests that some tumors benefit from repeated targeting of the same pathway. This observation is in line with the association between *KRAS *mutations and 12p gain in colorectal adenomas [10]. Recently, the "two-hit-hypothesis" was challenged in a study of inactivating *APC *gene mutations in colorectal cancer, in which three hits - including mutations, deletions, and copy number gain - were demonstrated in a subset of the tumors [11]. *APC *gene mutations, as well as *KRAS *mutations, are recognized as early events in colorectal cancer development. Based on our results and previous observations, concurrent *KRAS *mutations seem to occur in about 3% of colorectal cancers. The majority of these cases show coexisting mutations in codon 12, whereas a smaller number have mutations in different codons (12 and 13 or 12 and another codon) [8,12-15]. Different *KRAS *mutations have shown variable efficacy in downstream signaling transduction and also seem to involve different effector molecules [16,17]. The specific *KRAS *mutation may thereby influence different aspects of tumor progression, which could explain why repeated KRAS targeting is observed in a subset of colorectal cancer. Interestingly the p.Gly12Val mutation was found in 10 rectal cancers and 4 colon cancers, suggesting an overrepresentation in the former tumor type. This mutation has also been linked to a worse prognosis [16]. The addition of a p.Gly12Val mutation in the invasive component of a tumor already containing dual *KRAS *mutations (figure 1, case 3) suggests a role for repeated KRAS targeting during tumor progression and serves as a reminder to consider coexisting mutations when unexpected patterns occur in treatment predictive testing.

## Conclusion

In conclusion, our experience from predictive testing for *KRAS *mutations reveal a high mutation frequency (67%) in rectal cancers from females and thus implicates that this subset is the least likely to respond to anti-EGFR therapies. Concurrent *KRAS *mutations were observed in 3/136 tumors and although it remains a rare finding suggests that repeated *KRAS *targeting may occur during colorectal cancer progression.

## Competing interests

The investigators have received an unrestricted research grant and the principal investigator (M.N.) has in the past five years received lecture honorary from Merck A/S. No other competing interests apply.

## Authors' contributions

MJ carried out mutation analysis and drafted the manuscript. AE performed mutation analysis. TE, JE and DB identified and clinically managed patients and herein contributed with clinical data, DG selected tumor tissues, MN planned the study and was responsible for the clinical testing. All authors have contributed to and approved of the manuscript.

## Pre-publication history

The pre-publication history for this paper can be accessed here:


